# Limitation of Screening of Different Variants of SARS-CoV-2 by RT-PCR

**DOI:** 10.3390/diagnostics11071241

**Published:** 2021-07-12

**Authors:** Agathe Boudet, Robin Stephan, Sophie Bravo, Milène Sasso, Jean-Philippe Lavigne

**Affiliations:** 1 Virulence Bactérienne et Infections Chroniques, INSERM U1047, Université Montpellier, 30000 Nîmes, France; jean.philippe.lavigne@chu-nimes.fr; 2 Department of Microbiology and Hospital Hygiene, CHU Nîmes, 30000 Nîmes, France; robin.stephan@chu-nimes.fr (R.S.); milene.sasso@chu-nimes.fr (M.S.); 3 Department of Biochemistry, CHU Nîmes, 30000 Nîmes, France; sophie.bravo@chu-nimes.fr; 4 MIVEGEC, IRD, CNRS, Université Montpellier, 34000 Montpellier, France

**Keywords:** SARS-CoV-2, RT-PCR, screening method, variants

## Abstract

Since January 2021, the diffusion of the most propagated SARS-CoV-2 variants in France (UK variant 20I/501Y.V1 (lineage B.1.1.7), 20H/H501Y.V2 (lineage B.1.351) and 20J/H501Y.V3 (lineage P.1)) were urgently screened, needing a surveillance with an RT-PCR screening assay. In this study, we evaluated one RT-PCR kit for this screening (ID SARS-CoV-2/UK/SA Variant Triplex^®^, ID Solutions, Grabels, France) on 2207 nasopharyngeal samples that were positive for SARS-CoV-2. Using ID Solutions kit, 4.1% (92/2207) of samples were suspected to belonged to B.1.351 or P.1 variants. Next-generation sequencing that was performed on 67.4% (62/92) of these samples confirmed the presence of a B.1.351 variant in only 75.8% of the samples (47/62). Thirteen samples belonged to the UK variant (B.1.1.7), and two to A.27 with N501Y mutation. The thirteen with the UK variant presented one mutation in the *S-gene*, near the ΔH69/ΔV70 deletion (S71F or A67S), which impacted the detection of ΔH69/ΔV70 deletion. Using another screening kit (PKampVariantDetect SARS-CoV-2 RT-PCR combination 1 and 3^®^ PerkinElmer, Waltham, MA, USA) on the misidentified samples, we observed that the two mutations, S71F or A67S, did not impact the detection of the UK variant. In conclusion, this study highlights the limitations of the screening strategy based on the detection of few mutations/deletions as well as it not being able to follow the virus evolution.

## 1. Introduction

Since December 2019, a pandemic linked to a severe acute respiratory syndrome coronavirus 2 (SARS-CoV-2) caused pneumonia and severe acute respiratory syndrome worldwide [1,2]. From September 2020, new variants of concern (VOC) of SARS-CoV-2 were detected in Europe [3]. The first was the United Kingdom variant 20I/501Y.V1 (lineage B.1.1.7) [4], followed by two other lineages: the South-African variant 20H/H501Y.V2 (lineage B.1.351) [5] and the Brazilian variant 20J/H501Y.V3 (lineage P.1) [6,7]. Recently, other variants have emerged, such as the SARS-CoV-2 lineage A.27, which was firstly identified in Denmark in December 2020 but has its first origin in France [8]. Since January 2021, the propagation of these variants in France required an increased surveillance, based on RT-PCR screening assay, due to their important transmissibility and their potential to evade the host immune system [9]. Two of these lineages, B.1.351 and P.1, were of specific concern because they harbored the E484K mutation, which has been shown to enhance escape from neutralizing antibody inhibition in vitro [10], and may be associated with a reduced efficacy of the vaccine [11,12]. The aim of this study was to evaluate the main RT-PCR kit that is used for this screening: the ID SARS-CoV-2/UK/SA Variant Triplex^®^ (ID Solutions, Grabels, France).

## 2. Materials and Methods

### 2.1. Samples

This study was conducted at Nîmes University Hospital (France) between 27 January and 30 April 2021, commencing from the first nasopharyngeal screening performed. All admitted patients in our hospital were tested when they presented with potential COVID-19 related symptoms, including: fever, persistent cough, fatigue, myalgia, shortness of breath, diarrhoea, abdominal pain, chest pain, sore throat, loss of smell or taste; or when they were in close or prolonged contact with confirmed COVID-19 infected patients. For each patient, a nasopharyngeal swab was collected and sent to the Department of Microbiology that is accredited to perform RT-PCR assay for SARS-CoV-2. This retrospective study was approved by the local institutional review boards of the Nîmes University Hospital, France (IRB number: 20.05.01, approved on 4 May 2020). During this routine sample, we obtained a non-opposed consent of the patients to participate in SARS-CoV-2 studies. No data concerning patients and no follow-up were collected.

### 2.2. RT-PCR SARS-CoV-2 and Variants Detection

**Figure 1:**
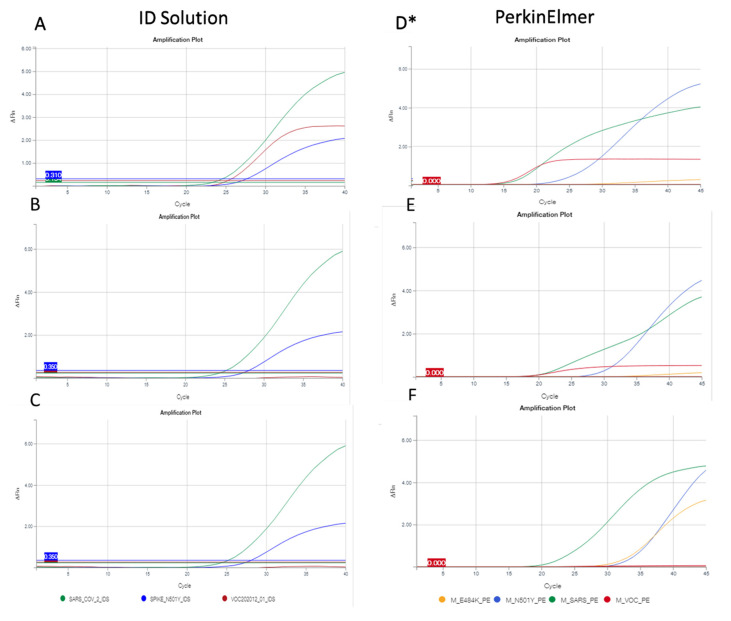

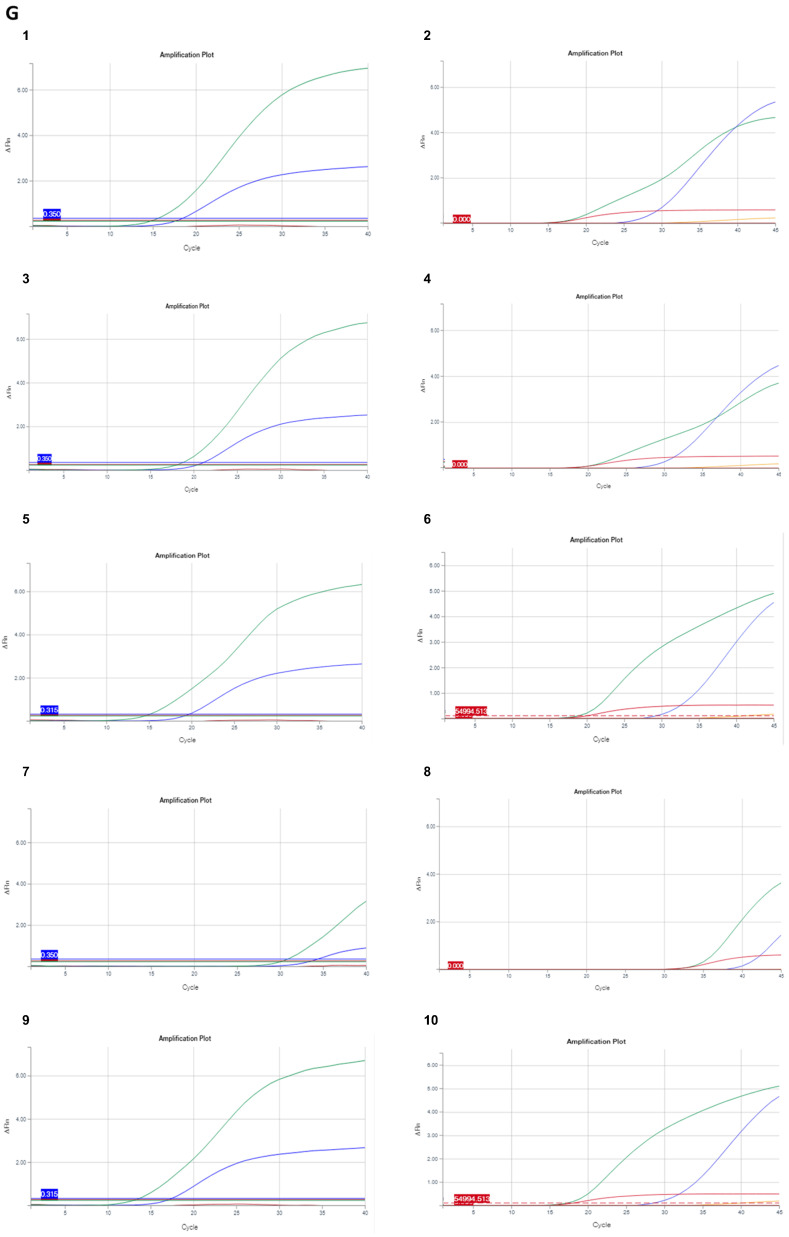

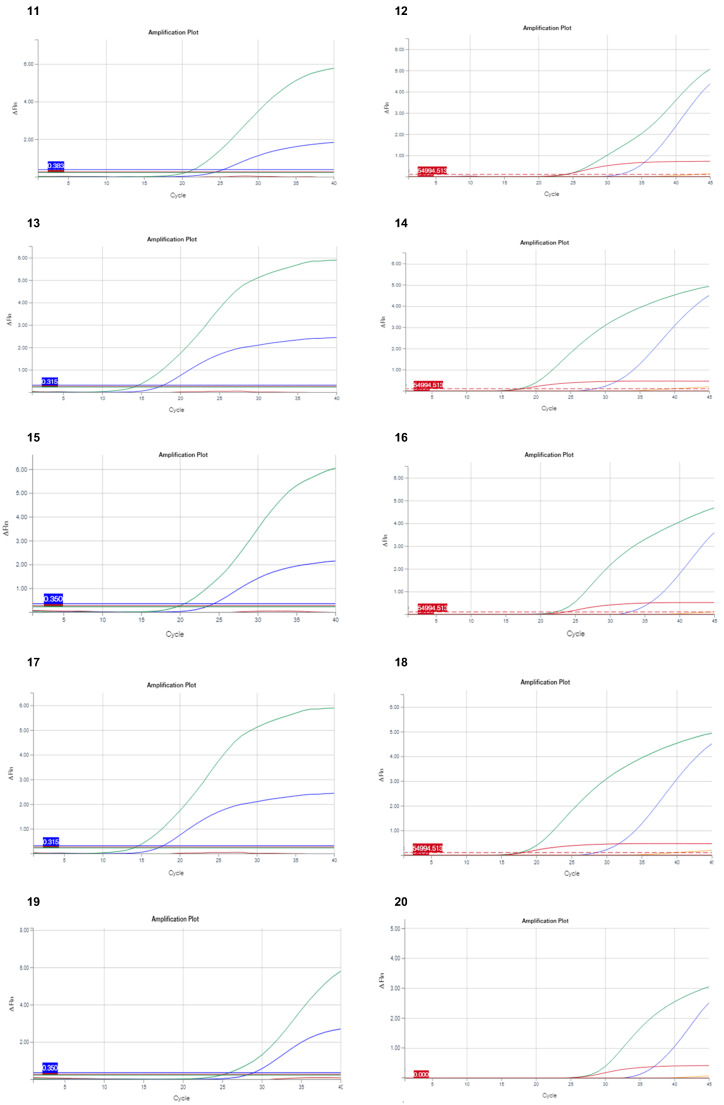

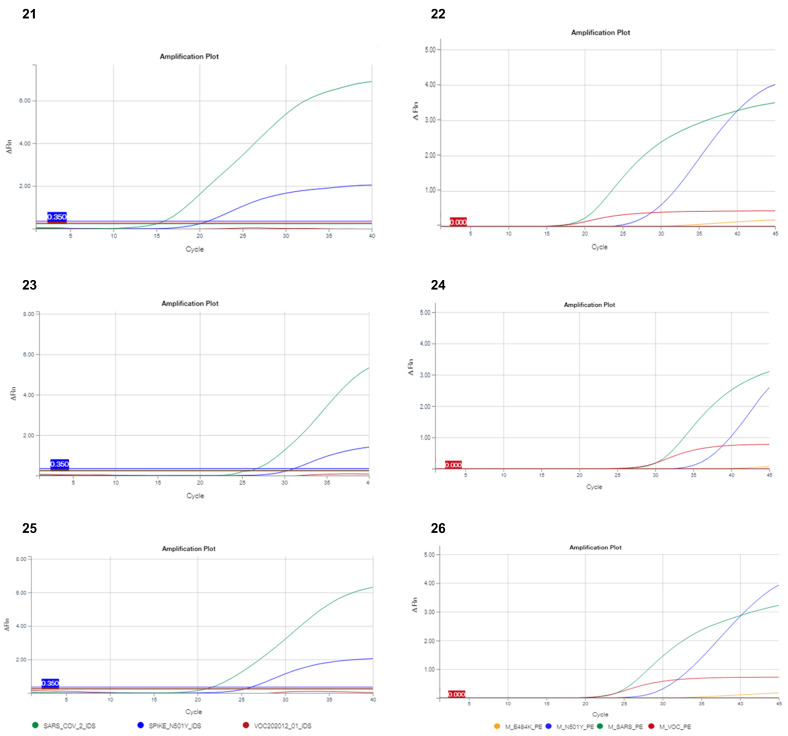
Representation of different variant profiles with the screening RT-PCR assays (on the left the results obtained with the ID Solution kit, and on the right those with PerkinElmer kit). All interpretations of described profiles were derived from NGS analysis. With the ID Solution kit, the green, blue and red curves correspond to SARS-CoV-2, N501Y and ΔH69/ΔV70, respectively. With the PerkinElmer kit, the green, blue, red and orange curves correspond to SARS-CoV-2, N501Y, ΔH69/ΔV7 and E484K, respectively. (**A**,**D**) profile of B.1.1.7 UK variant; (**B**,**E**) profile of B.1.1.7 variant with S71F mutation; (**C**,**F**) profile of P.1.351 or P.1 variants. * with or without S67A mutation, (**D**,**E**) were B.1.1.7 without E484K mutation. (**G**) detailed profiles of the 13 samples: 1 and 2: hCoV-19/France/OCC-NI-2104021309/2021; 3 and 4: hCoV-19/France/OCC-NI-2104041142/2021; 5 and 6: hCoV-19/France/OCC-NI-2103280925/2021; 7 and 8: hCoV-19/France/OCC-NI-2104011273/2021; 9 and 10: hCoV-19/France/OCC-NI-2103271225/2021; 11 and 12: hCoV-19/France/OCC-NI-2103301082/2021; 13 and 14: hCoV-19/France/OCC-NI-2103281047/2021; 15 and 16: hCoV-19/France/OCC-NI-2104091053/2021; 17 and 18: hCoV-19/France/OCC-NI-2103222347/2021; 19 and 20: hCoV-19/France/OCC-NI-2104270876/2021; 21 and 22: hCoV-19/France/OCC-NI-2104290828/2021; 23 and 24: hCoV-19/France/OCC-NI-2104262513/2021; 25 and 26: hCoV-19/France/OCC-NI-2104241354/2021.

RNA was extracted from clinical samples using the chemagic viral DNA/RNA kit special 96 on the chemagic platform (PerkinElmer, Waltham, MA, USA). The RT-PCR was performed using the kit SARS-CoV-2 *R-Gene*^®^ (bioMérieux, Marcy-l’Étoile, France), following the manufacturer’s recommendations. After this detection of the presence/absence of the virus, we screened the main variants that are circulating in Europe on positive specimens with cycle threshold (Ct) values lower than 35. This subsequent screening was performed with the ID SARS-CoV-2/UK/SA Variant Triplex^®^ kit (ID Solution, Grabels, France) [13]. This kit contained a positive control and a reaction mix with reverse transcriptase, Taq polymerase, primers and hydrolysis probes for the detection of SARS-CoV-2 targets, VOC202012/01 and 501Y.V2 variants. Assays were carried out with the following running conditions: 50 °C for 10 min, followed by 2 min at 95 °C, 40 cycles of 95 °C for 10 s and 65 °C for 30 s, according to manufacturer’s recommendations. Another screening kit (PKampVariantDetect SARS-CoV-2 RT-PCR combination 1 and 3^®^ (PerkinElmer)) was also performed retrospectively on the positive samples which belonged to non-UK variants, using the ID Solution kit [14]. All of these RT-PCR were conducted according to manufacturer’s instructions on the QuantStudio5 thermocycler (ThermoFisher Scientific, Waltham, MA, USA). The two screening kits presented a similar universal target of SARS-CoV-2 (*N/ORF1/ab-gene*), and two targets on the *S-gene*, the target of six nucleotides deletions (ΔH69/ΔV70) and the N501Y mutation. The Perkin Elmer RT-PCR kit had one more target for the E484K mutation, also on the *S-gene*. According to the kit used, three or four positive signals were present in the UK variant (Figure 1). Two or three positive targets that were composed by the universal target SARS-CoV-2 and the N501Y mutation +/− the E484K mutation suggested the presence of the variant 20H/501Y.V2 (B.1.351) or 20J/501Y.V3 (P.1). If the deletion of ΔH69/ΔV70 only was observed, the contamination might be due to a wild-type strain with a deletion, or another variant. The absence of the different variant targets (ΔH69/ΔV70, N501Y, E484K) was detected by the presence of the universal target of SARS-CoV-2 only and corresponded to a wild-type strain.

### 2.3. NGS Sequencing

Next generation sequencing (NGS) was performed on samples with a suspicion of B.1.351 and P.1 when Ct values were <30 on the first RT-PCR SARS-CoV-2 *R-Gene*^®^. Library preparation was performed with Ion AmpliSeq™ Library Kit Plus^®^ and Ion Torrent™ Dual Barcode Kit 1–96, according to the manufacturer’s recommendations (Ion torrent, ThermoFisher Scientific) [15]. Ion library Taqman quantitation kit^®^ was used to quantify and normalize the library. Sample libraries were pooled and processed using IonTorrent platform Ion S5™. CLC genomics (Qiagen, Hilden, Germany) was used for bioinformatics analysis, with genome reference Genbank MN908947.3. For a complete analysis and lineage determination, we employed two websites: Nextclade (https://clades.nextstrain.org/, accessed on 10 May 2021) and pangolineage (https://pangolin.cog-uk.io/, accessed on 10 May 2021).

## 3. Results

### 3.1. Prevalence of SARS-CoV-2 Variants

Over the studied period, 2207 samples were positive for SARS-CoV-2 from the same number of patients. Using the ID Solution kit screening, 71.8% of the samples (1585/2207) belonged to UK variant (B.1.1.7) (detection of the three targets SARS-CoV-2, N501Y and ΔH69/ΔV70, Figure 1A), and 4.1% (92 samples/2207) to variants B.1.351 or P.1 (detection of two targets SARS-CoV and 501Y, Figure 1C). The 530 remaining samples (24.1%) were other variants, without mutation N501Y nor deletion ΔH69/ΔV70 (e.g., lineages B1.177 or B1.160).

NGS was performed on 67.4% (62/92) of these samples that were suspected to belong to either B.1.351 or P.1 variants. The other 30 samples were not analysed because they were either included in intra-family or intra-institution clusters contaminations (13 cases/30), or harboured a Ct ≥30 cycles (17 cases/30).

After sequencing, 75.8% of the samples (47/62) belonged to lineage B.1.351, and no lineage P.1 was found. Among the 15 remaining samples, surprisingly, 13 were UK variants (B.1.1.7) and 2 belonged to lineage A.27 (19B/501Y) with a N501Y mutation. All of these 13 UK variants presented with one mutation in the *S-gene*, near the ΔH69/ΔV70 deletion: either S71F in position 21,774, or A67S in position 21,761 on the reference genome. These mutations were present in more than 99% of all samples (Table 1).

### 3.2. Problems in the Detection of Variants Using Multiplex RT-PCR

Using the ID Solution kit, the S71F and A67S mutations impacted the detection of ΔH69/ΔV70 deletion (Figure 1B). In all cases, NGS had definitively concluded that the samples belonged to the lineage B.1.1.7 (UK variant).

To investigate if another screening kit had the same problems in the detection of the UK variant, we performed the same screening on the 15 misidentified variants using the PerkinElmer kit. For samples with the S71F mutation, the fluorescence intensity of the ΔH69/ΔV70 target was lower (Figure 1E, red curve) than in B.1.1.7, without this mutation (Figure 1D). In the case of A67S mutations, we did not observe any impact on the detection of the UK variant (Figure 1D). In total, the PerkinElmer kit allowed for the identification of the UK variant, although the fluorescence was not equivalent to the positive control or the UK variant without the mutation S71F.

## 4. Discussion

The consequence of the pandemic SARS-CoV-2 is of concern, due to its high infectivity and fatality rate [16]. The RT-PCR screening that is performed on nasopharyngeal samples is a rapid and inexpensive strategy to characterize the main SARS-CoV-2 variants in a surveillance program to control the virus. This screening was mandatory in France since January 2021. Its implementation in numerous routine labs was simple, compared to that of full-genome sequencing, and has been largely deployed [13]. In our study, we have evaluated the commercialized screening kit that is mainly utilized. The ID Solution kit, performed prospectively in routine, has quickly highlighted the predominance of the UK variant, the most frequent variant currently isolated in France and Europe [13,17,18].

**Figure 2:**
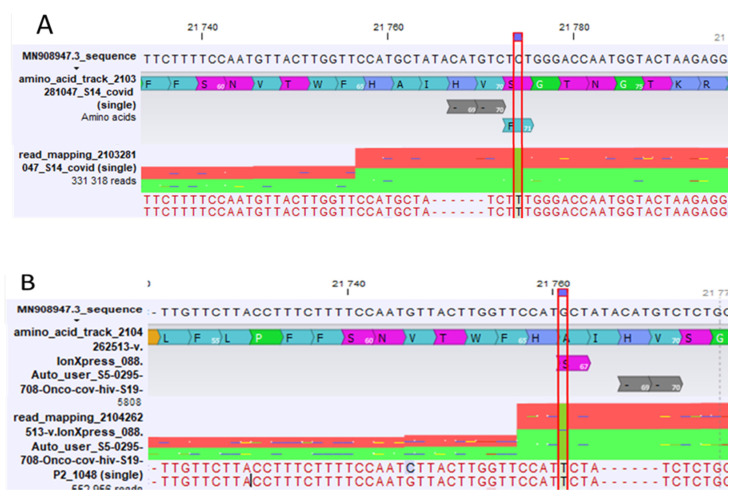
CLC genomics view of two mutations near the deletion ΔH69/ΔV70. (**A**) mutation view of S71F; (**B**) mutation view of A67S.

When the RT-PCR using the ID Solution kit detected two targets (universal SARS-CoV-2 (*N-gene*) and N501Y (*S-gene*)), the manufacturer proposed to conclude that there is the presence of lineage B.1.351; however, the N501Y mutation is present in many variants (e.g., lineage A.27 [19]). Our study supports the interest in performing a full-genome sequencing in these cases. Indeed, among the 62 cases of suspected lineage B.1.351 or P.1, we confirmed the misidentification of 15 samples (24.2%) that belonged to lineages B.1.1.7 (21%) and A.27 (3.2%). Among these samples, we failed to detect two interesting samples belonging to the UK variant (B.1.1.7). Indeed, the SARS-CoV-2 genome sequencing of these samples highlighted the presence of two mutations, S71F and A67S, localized very closely to the targeted deletion (Figure 2), which could explain the lack of detection. Using the PerkinElmer kit, we observed a low fluorescence intensity in this targeted deletion (Figure 1E), but only in samples with the S71F mutation. The difference between the two screening kits used in this study was the hybridization temperature. This temperature was lower with the PerkinElmer kit (62 °C) than with the ID Solution kit (65 °C). Thus, we suspect that the PerkinElmer probes can bind more easily to the sequence genome, despite the presence of the S71F mutation. However, this binding was hindered by the mutation and the amplification curve, which corresponded to a weaker fluorescence signal than expected. Moreover, we can hypothesize that the sequences of the PCR primers are different, even if they are not known by users, as we can detect the A67S mutation with the PerkinElmer kit. Our results suggest that a heightened attention of the virologist is needed during the result interpretation.

Recent studies have evaluated different screening kits in comparison to NGS. The authors demonstrated a high concordance between the two methods, reinforcing the interest of screening methods [20,21,22,23,24]. Here, we showed that the PerkinElmer kit performed better than the ID Solution kit, avoiding the problem of mutation detection, notably the detection of E484K mutation. This mutation is always present in the lineages B.1.351 and P.1, but also in the UK variant [25]. The detection of the E484K mutation has a clinical importance because it is associated with a reduced efficacy of the vaccine, or confers resistance to monoclonal antibodies or convalescent plasma [10,11,12]. However, even if the PerkinElmer kit presented better performance in our study, we suggest that a similar problem of detection could be observed if a mutation was localized closed to the E484K target, impacting on the two RT-PCR screening kits assays. Given the number of mutations present in the different VOCs and the constant appearance of new variants of interest (VOI), we assume that these screenings methods targeting few mutations are less adapted to the evolution of the COVID-19 epidemic [25].

Currently, despite the obligation of Direction Générale de la Santé (DGS) in France, the screening of the UK variant, in view of this prevalence, is no longer necessary for diagnosis or clinical impact [26]. With the evolution of VOCs and VOIs, the French recommendations evolved on 26 May 2021 to screen only E484K and two other mutations (E484Q, L452R), with an impact similar to E484K. It is important to know that rapid screening tools seem limited to follow the evolution of the virus and detect the different mutations [8,25,27]. Although NGS is more expensive, time consuming, and needs adapted structures with technical and biological experience, this technology is the unique tool able to determine the different variants and follow the evolution of SARS-CoV-2.

## Figures and Tables

**Table 1:** Details of the characteristics of the NGS sequences deposited in GISAID (Global Initiative on Sharing All Influenza Data).

Mutation	Virus Name Gisaid	Number GISAID	Coverage	Coverage (%)
S71F	hCoV-19/France/OCC-NI-2104021309/2021	EPI_ISL_1972901	5835	5179/5179 (100%)
	hCoV-19/France/OCC-NI-2104041142/2021	EPI_ISL_1972902	5867	1558/1559 (99.9%)
	hCoV-19/France/OCC-NI-2103280925/2021	EPI_ISL_1972903	3039	1879/1882 (99.9%)
	hCoV-19/France/OCC-NI-2104011273/2021	EPI_ISL_1915508	4574	3247/3250 (99.9%)
	hCoV-19/France/OCC-NI-2103271225/2021	EPI_ISL_1915509	6630	6049/6060 (99.8%)
	hCoV-19/France/OCC-NI-2103301082/2021	EPI_ISL_1972904	5392	2602/2604 (99.9%)
	hCoV-19/France/OCC-NI-2103281047/2021	EPI_ISL_1972905	2468	1737/1740 (99.9%)
	hCoV-19/France/OCC-NI-2104091053/2021	EPI_ISL_1915510	7209	6393/6398 (99.9%)
	hCoV-19/France/OCC-NI-2103222347/2021	EPI_ISL_1524908	3770	3268/3269 (99.9%)
	hCoV-19/France/OCC-NI-2104270876/2021	EPI_ISL_2131449	5376	5866/5873 (99.9%)
	hCoV-19/France/OCC-NI-2104290828/2021	EPI_ISL_2131450	7257	6255/6258 (99.9%)
A67S	hCoV-19/France/OCC-NI-2104262513/2021	EPI_ISL_2131448	4244	5229/5242 (99.7%)
	hCoV-19/France/OCC-NI-2104241354/2021	EPI_ISL_2131447	3690	3123/3126 (99.9%)

## Data Availability

Not applicable.
